# Recurrent Rare Copy Number Variants Increase Risk for Esotropia

**DOI:** 10.1167/iovs.61.10.22

**Published:** 2020-08-11

**Authors:** Mary C. Whitman, Silvio Alessandro Di Gioia, Wai-Man Chan, Alon Gelber, Brandon M. Pratt, Jessica L. Bell, Thomas E. Collins, James A. Knowles, Christopher Armoskus, Michele Pato, Carlos Pato, Sherin Shaaban, Sandra Staffieri, Sarah MacKinnon, Gail D E. Maconachie, James E. Elder, Elias I. Traboulsi, Irene Gottlob, David A. Mackey, David G. Hunter, Elizabeth C. Engle, for the Strabismus Genetics Research Consortium

**Affiliations:** 1 Department of Ophthalmology, Boston Children's Hospital, Boston, Massachusetts, United States; 2 Department of Ophthalmology, Harvard Medical School, Boston, Massachusetts, United States; 3 F.M. Kirby Neurobiology Center, Boston Children's Hospital, Boston, Massachusetts, United States; 4 Department of Neurology, Boston Children's Hospital, Boston, Massachusetts, United States; 5 Department of Cell Biology, SUNY Downstate Health Sciences University, Brooklyn, New York, United States; 6 Institute for Genomic Health, SUNY Downstate Medical Center, Brooklyn, New York, United States; 7 Present address: Department of Pathology and ARUP Laboratories, University of Utah School of Medicine, Salt Lake City, Utah, United States; 8 Centre for Eye Research Australia, Royal Victorian Eye and Ear Hospital, East Melbourne, Victoria, Australia; 9 Department of Neuroscience, Psychology and Behavior, The University of Leicester Ulverscroft Eye Unit, University of Leicester, Leicester, United Kingdom; 10 Department of Ophthalmology, Royal Children's Hospital, University of Melbourne, Parkville, Victoria, Australia; 11 Department of Pediatrics, The University of Melbourne, Parkville, Victoria, Australia; 12 Department of Pediatric Ophthalmology and Strabismus, Cole Eye Institute, Cleveland Clinic, Cleveland, Ohio, United States; 13 Centre for Ophthalmology and Visual Science, Lions Eye Institute, University of Western Australia, Perth, Australia; 14 Menzies Institute for Medical Research, University of Tasmania, Hobart, Australia; 15 Centre for Eye Research Australia, University of Melbourne, Melbourne, Australia; 16 Department of Neurology, Harvard Medical School, Boston, Massachusetts, United States; 17 Howard Hughes Medical Institute, Chevy Chase, Maryland, United States

**Keywords:** esotropia, strabismus, copy number variant, CNV, genetics of strabismus

## Abstract

**Purpose:**

To determine whether rare copy number variants (CNVs) increase risk for comitant esotropia.

**Methods:**

CNVs were identified in 1614 Caucasian individuals with comitant esotropia and 3922 Caucasian controls from Illumina SNP genotyping using two Hidden Markov model (HMM) algorithms, PennCNV and QuantiSNP, which call CNVs based on logR ratio and B allele frequency. Deletions and duplications greater than 10 kb were included. Common CNVs were excluded. Association testing was performed with 1 million permutations in PLINK. Significant CNVs were confirmed with digital droplet polymerase chain reaction (ddPCR). Whole genome sequencing was performed to determine insertion location and breakpoints.

**Results:**

Esotropia patients have similar rates and proportions of CNVs compared with controls but greater total length and average size of both deletions and duplications. Three recurrent rare duplications significantly (*P* = 1 × 10^−^^6^) increase the risk of esotropia: chromosome 2p11.2 (hg19, 2:87428677-87965359), spanning one long noncoding RNA (lncRNA) and two microRNAs (OR 14.16; 95% confidence interval [CI] 5.4–38.1); chromosome 4p15.2 (hg19, 4:25554332-25577184), spanning one lncRNA (OR 11.1; 95% CI 4.6–25.2); chromosome 10q11.22 (hg19, 10:47049547-47703870) spanning seven protein-coding genes, one lncRNA, and four pseudogenes (OR 8.96; 95% CI 5.4–14.9). Overall, 114 cases (7%) and only 28 controls (0.7%) had one of the three rare duplications. No case nor control had more than one of these three duplications.

**Conclusions:**

Rare CNVs are a source of genetic variation that contribute to the genetic risk for comitant esotropia, which is likely polygenic. Future research into the functional consequences of these recurrent duplications may shed light on the pathophysiology of esotropia.

Strabismus affects 2% to 4% of the population and causes amblyopia, loss of binocular vision, and lower quality of life.^1^^,^^2^ Strabismus runs in families, and population, family, and twin studies support a genetic contribution.^3^^−^^5^ Twin meta-analysis supports a strong genetic contribution,^3^ particularly for esodeviations.^6^ The relative risk for first-degree relatives of an affected proband is estimated to be between 3 and 5.^3^^,^^5^^,^^7^^−^^9^ The heritability factor remains significant after correction for the known environmental risk factors:^5^ low birth weight, prematurity, maternal smoking, and advanced maternal age.^10^^–^^17^

Causative genes have been identified for paralytic strabismus syndromes, in which patients cannot fully move their eyes.^18^ In common forms of strabismus, however, no specific mutations have been reported, despite reported mapping of three Mendelian loci (7p22.1, 4q28.3 and 7q31.2).^7^^,^^19^^,^^20^ We recently completed a genome wide association study (GWAS) of non-accommodative esotropia and identified one risk allele, an intronic single nucleotide polymorphism (SNP) of the *WRB* gene, which affects expression of *WRB* and neighboring genes.^21^ A second GWAS, using self-reported strabismus in the UK Biobank, identified a locus on chromosome 17q25, which extends across the NPLOC4-TSPAN10-PDE6G gene cluster.^22^ This locus has been associated through GWAS with several eye conditions, including macular thickness,^23^ astigmatism,^24^ retinal microvascular size,^25^ and myopia.^26^

Genetic variation can result from DNA sequence differences, duplications or deletions of genomic elements (copy number variants [CNVs]), or complex genetic rearrangements. CNVs can alter gene function, gene dosages, regulatory elements, or 3D chromatin structure.^27^ CNVs have been implicated in neurodevelopmental disorders with complex inheritance, including autism spectrum disorder,^28^^–^^37^ intellectual disabiltity,^38^^–^^42^ and Tourette syndrome.^43^ Strabismus is a neurodevelopmental disorder affecting the neural pathways that control ocular alignment and binocular fusion and is prevalent in patients with other neurodevelopmental disorders. We therefore examined our cohort of individuals with isolated esotropia for rare CNVs. We report here on three rare, recurrent DNA duplications that increase the risk of esotropia.

## Methods

This study was approved by the local Institutional Review Boards of Boston Children's Hospital, Boston, MA, USA; The Cleveland Clinic, Cleveland, OH, USA; Leicestershire, Northamptonshire and Rutland Committee for the National Research Ethics Service, UK; Rutland Research Ethics Committee, UK; Human Research Ethics Committee, Royal Victorian Eye and Ear Hospital, East Melbourne, Victoria, Australia; Princess Margaret Hospital, Perth, Western Australia; and Sir Charles Gairdner Hospital, Perth, Western Australia. Informed consent was obtained from all participants. All investigations were conducted in accordance with the principles of the Declaration of Helsinki.

### Cases

The esotropia cohort consists of patients from our previous GWAS,^21^ including both accommodative and nonaccommodative cases. Inclusion and exclusion criteria were the same: manifest or intermittent esotropia of any size, a history of strabismus surgery for comitant esotropia, or esophoria >10 prism diopters. Accommodative esotropia was defined as manifest esotropia that reduced with hyperopic correction to <10 prism diopters. Infantile esotropia was defined as esotropia with onset before the age of 12 months. Nonaccommodative esotropia was defined as manifest or intermittent esotropia with onset after age 12 months that did not reduce to <10 prism diopters with hyperopic correction; this includes partially accommodative esotropia. By definition, fully accommodative cases did not have strabismus surgery; any patient who had strabismus surgery was classified as either non-accommodative or infantile, depending on age of onset. Exclusion criteria included structural ocular abnormality causing acquired vision loss; structural brain abnormality on neuroimaging; deprivation amblyopia; molecularly defined genetic syndromes or diagnoses associated with strabismus, such as trisomy 21 or craniosynostosis; or defined nonheritable cause of strabismus. A total of 2030 participants who self-reported as White of European ancestry (and in whom principal component analysis confirmed European ancestry) were enrolled: 1105 from Boston Children's Hospital, 745 from Australia (private ophthalmologists and public hospitals in Victoria, Western Australia, Tasmania, and New South Wales), 111 from Leicester, University Hospitals of Leicester, UK, 52 from Cole Eye Institute (Cleveland Clinic), 5 from Children's Hospital of Philadelphia, and 12 self-referred. After all quality control filters for CNV calling, the total number of participants included was 1614.

### Controls

Control subjects of Caucasian ancestry were ascertained in which participants were genotyped on the Illumina Omni platform, and intensity data were available. This included controls from the Genomic Psychiatry Cohort and publicly-available controls from a GWAS of Fuchs’ Endothelial Corneal Dystrophy (FECD) (accession number: phs000421.v1.p1), derived from the database of Genotypes and Phenotypes (dbGaP). After all quality control filters, 3922 control participants were included. None of the controls were reported to have strabismus, although strabismus was not specifically excluded from the ascertainment cohorts.

### Genotyping

Esotropia patients were genotyped on Illumina Infinium human OmniExpress-24v1-0 array. Control cohorts from FECD were genotyped on Illumina HumanOmni 2.5 Versions 4v_1H array and the Genomic Psychiatry cohort was genotyped on Illumina OmniExpress 12v1.0. 98% of the individual SNPs present on OmniExpress-24v1-0 are present on the other arrays. SNP clustering and genotype calling was performed with GenomeStudio v2.0 (Illumina, San Diego, CA, USA). Samples with a call rate <0.98 or with discordant sex were excluded.

### Intensity Sample Quality Control

Intensity-based metrics were used to eliminate samples unsuitable for CNV calling. These included the following: waviness factor (WF)—a measure of the waviness in intensity values, a known artifact caused by improper DNA concentration that can lead to spurious calls; Log-R ratio standard deviation (LRR_SD)—a measure of the overall variance in intensity; B allele frequency drift (BAF_DRIFT)—a summary of the deviation of BAF from expected values. Cutoff values for each were determined empirically. Samples included had LRR-SD of <0.3, absolute value of WF <0.43, and BAF_DRIFT <0.01. We eliminated samples with greater than 50 CNV calls, because those are more likely to be spurious calls. The final samples included 1614 esotropia patients and 3922 controls.

### CNV Calling

We used two hidden Markov Model (HMM)–based CNV calling algorithms, PennCNV^44^^,^^45^ (version 1.0.4) and QuantiSNP^46^ (version 2). These algorithms detect CNVs based on B allele frequency (BAF) and logR ratio (LRR). We created GC wave–adjusted LRR intensity files for all samples using PennCNV's *genomic_wave.pl* script.^47^ Because HMM algorithms can artificially break up large CNVs, CNV segments were merged using PennCNV's *clean_cnv.pl* script if they were of the same copy number and the intervening markers were less than 20% of the total of both segments. Calls from the two programs were merged by taking the intersection of overlapping calls of the same copy number. Only CNVs called by both programs, greater than 10kb, and encompassing 10 or more SNPs were included in the final call set.

### Call Filtering

CNVs were filtered out if they overlapped (>50%) with regions known to generate artifacts in SNP-based CNV detection: immunoglobulin domain regions, segmental duplications, telomeric ends and centromeric regions. We eliminated deletions with a PennCNV confidence score <25 and duplications with a PennCNV confidence score <10. These cutoffs were determined empirically based on confirmation of CNVs using ddPCR. Several deletions with confidence scores <25 were not confirmed by ddPCR, but duplications with scores above 10 were confirmed. To limit our dataset to rare CNVs, we eliminated CNVs that overlapped (>50%) with common CNVs (any CNV with >10% prevalence in large studies compiled by the DECIPHER database or Database of Genetic Variants (DGV)). We further eliminated any CNVs present in greater than 1% of the controls used in this study.

### CNV Annotation

Rare CNVs were annotated for gene content according to RefSeq for the hg19 assembly using PennCNV.

### Association Testing

We performed 1 × 10^6^ label-swapping permutations in PLINKv1.07 to determine both locus-specific and genome-wide *P* values empirically, using the max(T) method.^48^ In the segmental test, case and control frequencies were calculated at each unique CNV breakpoint. For the gene-based test, frequencies were based on the number of genic CNVs at each gene locus. Association testing was conducted separately for deletions and duplications.

### Removal of Related Individuals

Because recruitment of the esotropia cohort focused on patients with affected relatives, some of the participants were related. To ensure this was not biasing the results, we repeated association testing and per-gene testing after removing related individuals. From each family, the individual with the highest quality control scores was included, and others were excluded. After removing related individuals, 1379 unrelated esotropic individuals remained.

### Confirmation of CNVs

Significant CNVs were confirmed using digital droplet PCR (ddPCR, BioRad).^49^ Probes were designed using Bio-Rad's proprietary algorithm, and the assay was performed in duplicate for each patient. Locations of the probes were hg19|chr2:87790100-87790222, hg19|chr4:25561415-25561537, and hg19|chr10:47100043-47100165.

### Determination of Insertion Sites

Whole-genome sequencing (WGS) was performed for three individuals with each of the significant CNVs to confirm the presence of CNVs and determine the insertion sites and breakpoints. WGS was performed at the Broad Institute of MIT and Harvard and called against the hg38 reference genome, which is a single representation of multiple genomes. Results were interpreted by examining read depth and split reads at the identified areas using integrated genome viewer software and compared to other individuals sequenced in the same call set.

## Results

### Esotropia Cohort

Of the 2030 individuals with esotropia included in the previous GWAS,^21^ 1614 passed quality control measures for CNV calling. This included 851 females and 763 males; 911 from the US, 84 from the UK, and 620 from Australia. A total of 224 had accommodative esotropia, 317 had infantile esotropia, and 1075 had nonaccommodative esotropia.

### Rare CNV Burden

**Figure 1.**
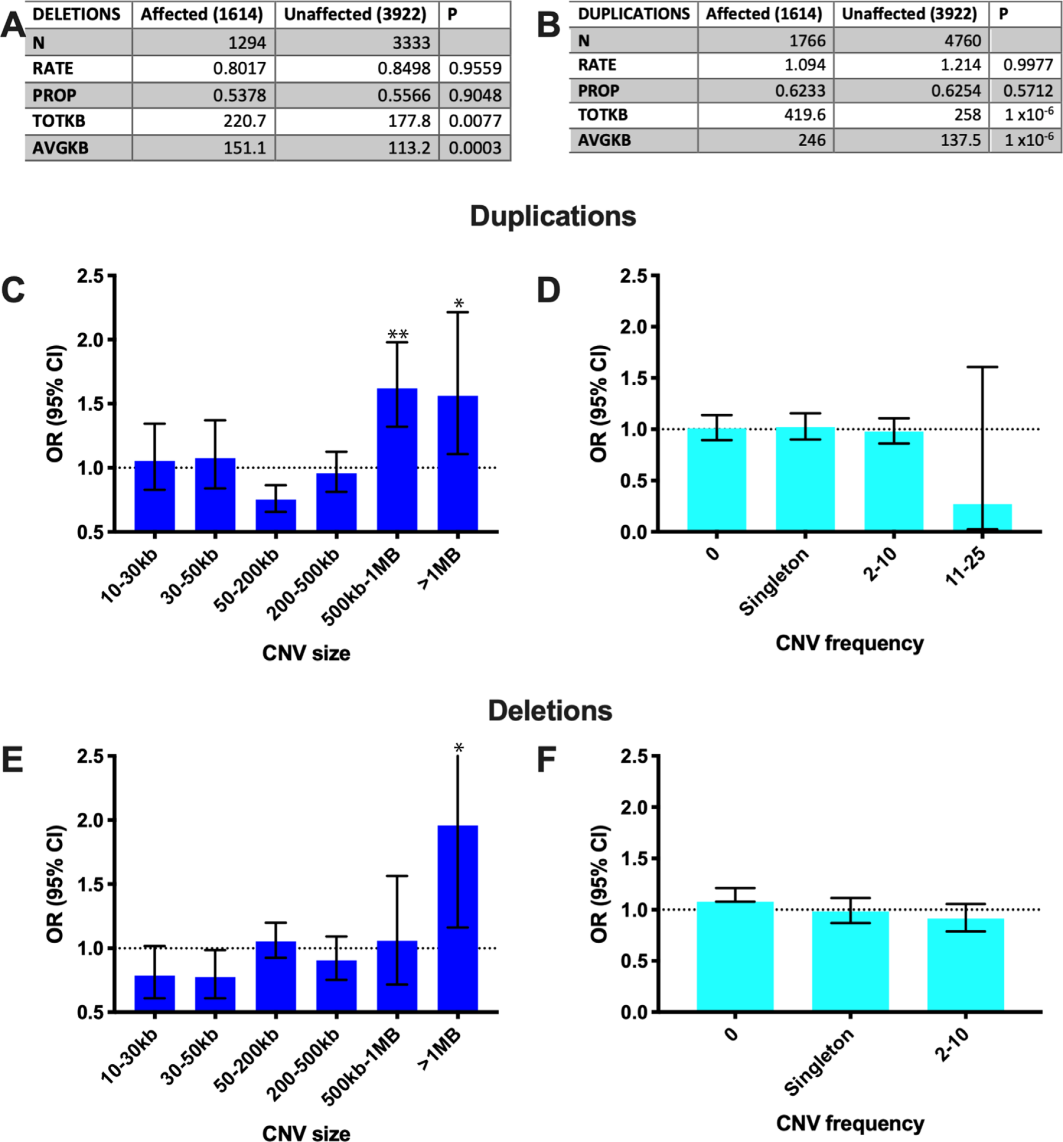
Rare CNV burden in esotropia cases and controls. Esotropia cases have similar rates (number per person) and proportions (percent of people with at least one) of rare (<1% frequency), >10 kb deletions (**A**) and duplications (**B**) to controls. The total length and average size of each CNV, however, are larger in esotropia cases. (**C–F**) Odds ratios for esotropia given different CNV sizes (**C**, duplications, **E**, deletions) and frequencies (**D**, duplications, **F**, deletions). Duplications of 500 kb–1 MB and greater than 1 MB and deletions >1 MB were associated with higher risk of esotropia. Frequency of CNVs was not associated with esotropia. * *P* < 0.015, ** *P* < 0.0001.

Esotropia patients and controls have similar rates of rare CNVs, with approximately 0.8 deletions and 1.1 duplications per person. Similar proportions have at least one rare deletion (∼54%) or duplication (∼62%). Esotropia patients have a greater total CNV length: an average of 220.7KB of total (rare) deletions, versus 177.8KB in controls (*P* = 0.007) and 419.6KB of total (rare) duplications versus 258KB in controls (*P* = 1 × 10^−^^6^). The average size of each individual CNV was larger in esotropia patients: deletions averaged 151.1 KB versus 113.2 KB in controls (*P* = 0.0003) and duplications averaged 246 KB versus 137.5 KB in controls (*P* = 1 × 10^−^^6^) (Figs. 1A, 1B). We partitioned across CNV size and frequency and calculated odds ratios. Esotropia patients were more likely to have a total duplication burden of 500kb-1MB (OR 1.6, 95% confidence interval [CI] 1.3–1.98, *P* < 0.0001) and >1 MB (odds ratio [OR] 1.6, 95% CI 1.1–2.2, *P* = 0.0109) (Fig. 1C) or a total deletion burden >1MB (OR 1.96, 95% CI 1.16–3.32, *P* = 0.013; Fig. 1E). When combining deletions and duplications, esotropia cases were more likely to have a total CNV burden of 500 kb to 1 MB (OR 1.3, 95% CI 1.1–1.5, *P* = 0.0033) or >1 MB (OR 1.8, 95% CI 1.4–2.4, *P* < 0.0001). Esotropia patients showed no increase in frequency (number) of duplications (Fig. 1D), deletions (Fig. 1F), or total CNVs.

### Three Rare Recurrent Duplications Confer Risk for Esotropia

**Figure 2.**
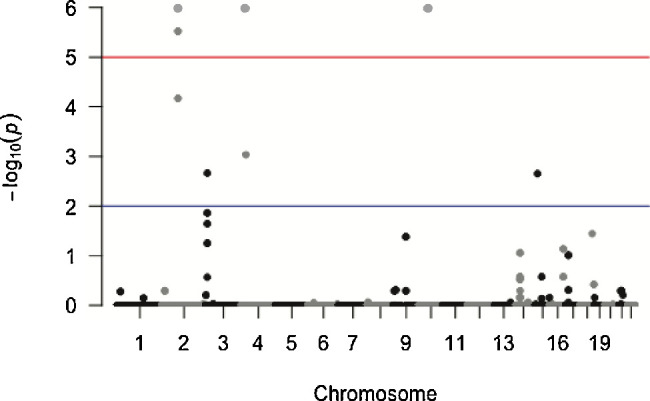
Segmental tests show three significant duplications. Manhattan plot of segmental association test results representing genome-wide corrected p values calculated at each CNV breakpoint. *Black circles* represent deletions, and *gray circles* represent duplications. Three duplications, on chromosomes 2, 4, and 10, were significant to *P* = 1 × 10^−6^. The three *circles* for chromosome 2 represent the same duplication, which has different breakpoints in different individuals. Some of the individual breakpoints have lower *P* values.

To test for enrichment of rare CNVs at individual loci, we conducted a segmental genome-wide association test, treating deletions and duplications separately. We also conducted a complementary gene-based test, conditioned on CNVs affecting exons, to account for potentially non-overlapping CNVs affecting the same gene. In CNV analysis, in contrast to SNP-based GWAS, there is no established *P* value threshold for genome-wide significance. Therefore we established locus-specific and genome-wide corrected *P* values empirically through 1,000,000 label-swapping permutations, using the max(T) method,^48^ following the methods of Huang et. al.^43^ Association testing identified three recurrent rare duplications enriched among esotropia patients that survived genome-wide correction for multiple testing (Fig. 2). No specific deletions reached genome-wide significance.

**Figure 3.**
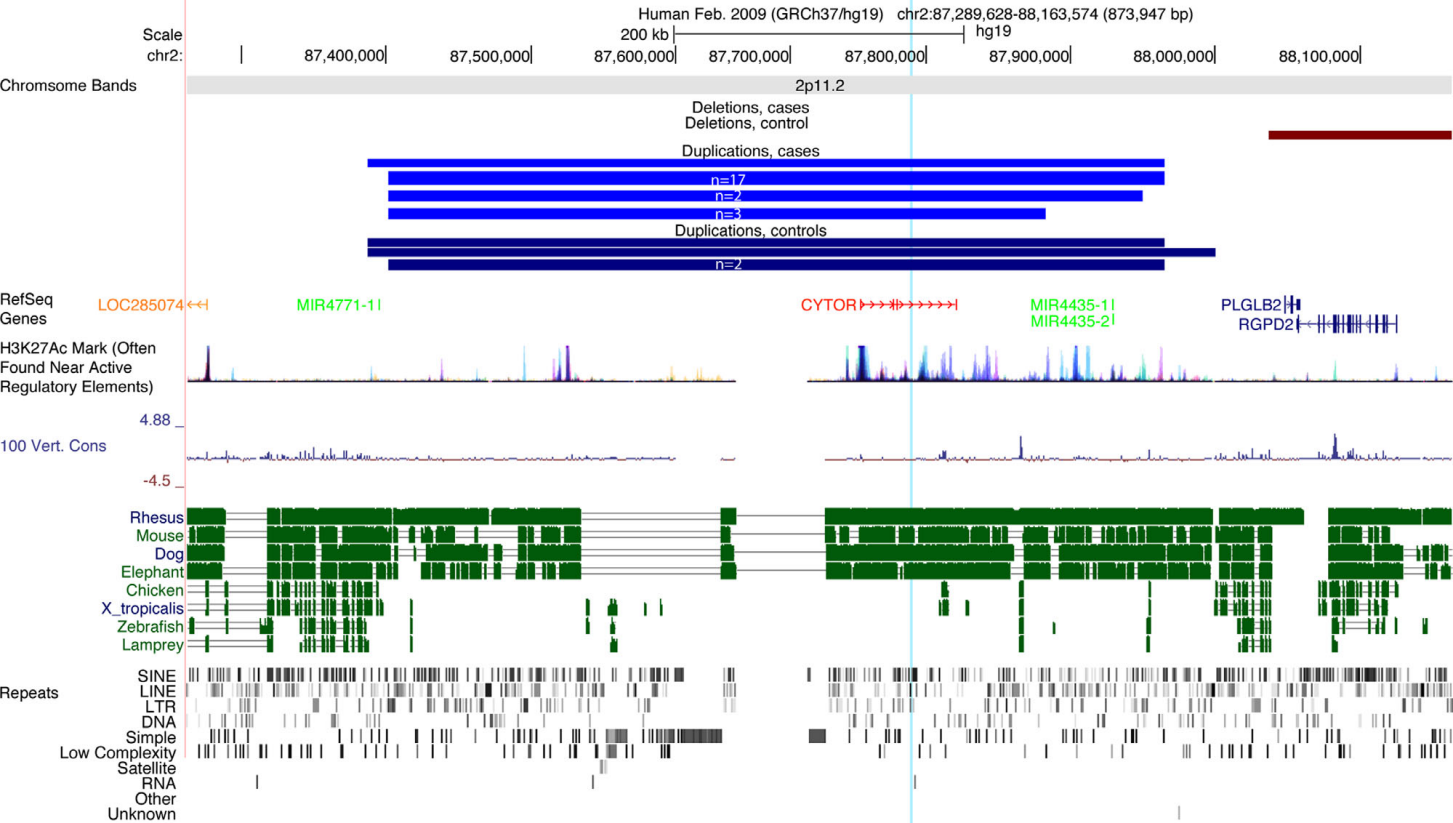
Chromosome 2 duplication significantly enriched in esotropia cases. UCSC genome browser plot showing the region of duplication on 2p11.2 (2:87428677-87965359). Duplications across this region were present in 23 cases (*light blue*, n indicates number with each set of breakpoints) and four controls (*dark blue*). A nearby deletion was present in one control (*dark red*). RefSeq genes are listed underneath. Protein coding genes are denoted in *blue*, lncRNA genes in *red*, micro RNAs in *green*, and noncoding RNAs in *orange*. H3K27Ac mark indicates several putative regulatory regions fall within the duplication. This area is not well conserved over 100 vertebrates, but the genic and putative regulatory regions are well conserved in primates and other mammals. The *blue vertical line* indicates the position of the ddPCR probe used to confirm the duplication. The gap in the annotations indicates an unmappable area of the reference genome, usually because it is highly repetitive or of low complexity. At bottom are indicated repeats in the region identified by RepeatMasker: SINE, short interspersed nuclear elements; LINE, long interspersed nuclear elements; LTR, long terminal repeat elements; DNA, DNA repeat elements; SIMPLE, microsatellites, low complexity repeats, satellite repeats, RNA repeats, and other repeats.

Association testing identified a significant 536kb locus on chromosome 2p11.2 (hg19, chr2:87428677-87965359, p_corr_ = 1 × 10^−^^6^), spanning the long noncoding RNA (lncRNA) *CYTOR*, and overlapping microRNAs miR4435-1 and miR4435-2, which were also identified with the gene-based test (p_corr_ = 1 × 10^−^^6^, 3 × 10^−^^6^, and 3 × 10^−^^6^, respectively). This CNV was present in 23 cases (1.4%) and four controls (0.1%), corresponding to a substantially increased esotropia risk (OR 14.16 (95%CI 5.4-38.1)). This region contains several putative regulatory regions and has areas with conservation among mammals but not other vertebrates (Fig. 3).

**Figure 4.**
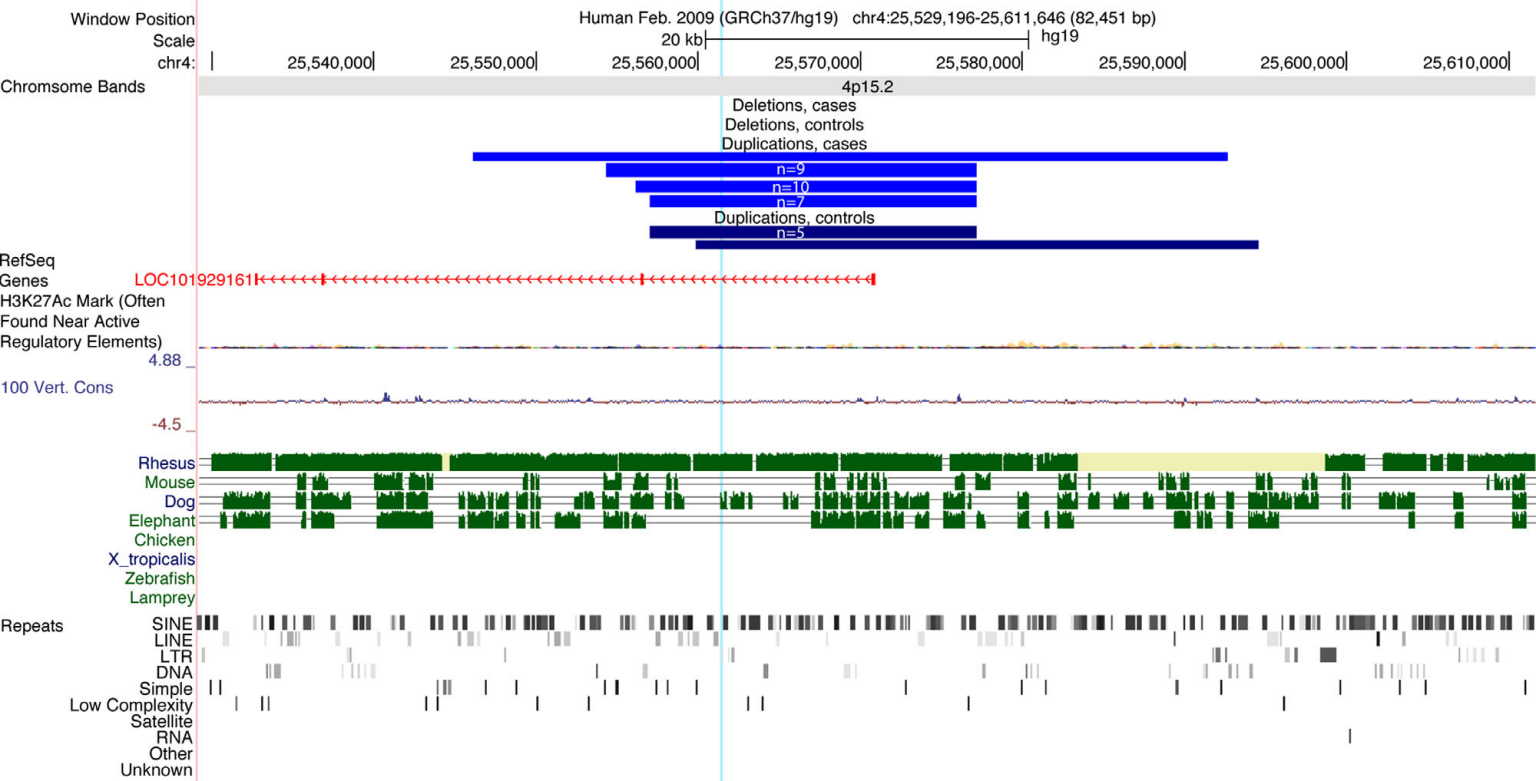
Chromosome 4 duplication significantly enriched in esotropia cases. UCSC genome browser plot showing the region of duplication on 4p15.2 (4:25554332-25577184). Duplications across this region were present in 27 cases (*light blue*, n indicates number with each set of breakpoints) and six controls (*dark blue*). The duplication includes exon 1 of LOC101929, a long noncoding RNA (*orange*). H3K27Ac marks show no putative regulatory regions within the duplication. This area is well conserved with monkeys, but not other mammals. There is no conservation with nonmammals. The *blue vertical line* indicates the position of the ddPCR probe used to confirm the duplication. At bottom are indicated repeats in the region identified by RepeatMasker: SINE, short interspersed nuclear elements; LINE, long interspersed nuclear elements; LTR, long terminal repeat elements; DNA, DNA repeat elements; SIMPLE, microsatellites, low complexity repeats, satellite repeats, RNA repeats, and other repeats.

A significant 22.8kb locus was identified on chromosome 4p15.2 (hg19, chr4:25554332-25577184, p_corr_ = 1 × 10^−^^6^), spanning exon 1 of the lncRNA LOC101929161, which was also identified with the gene-based test (p_corr_ = 1 × 10^−^^6^). A CNV in this location was present in 27 cases (1.7%) and six controls (0.2%), corresponding to a substantially increased esotropia risk (OR 11.1 (95%CI 4.6-25.2). This region does not contain any putative regulatory elements and shows conservation with monkeys but not with other animals (Fig. 4).

**Figure 5.**
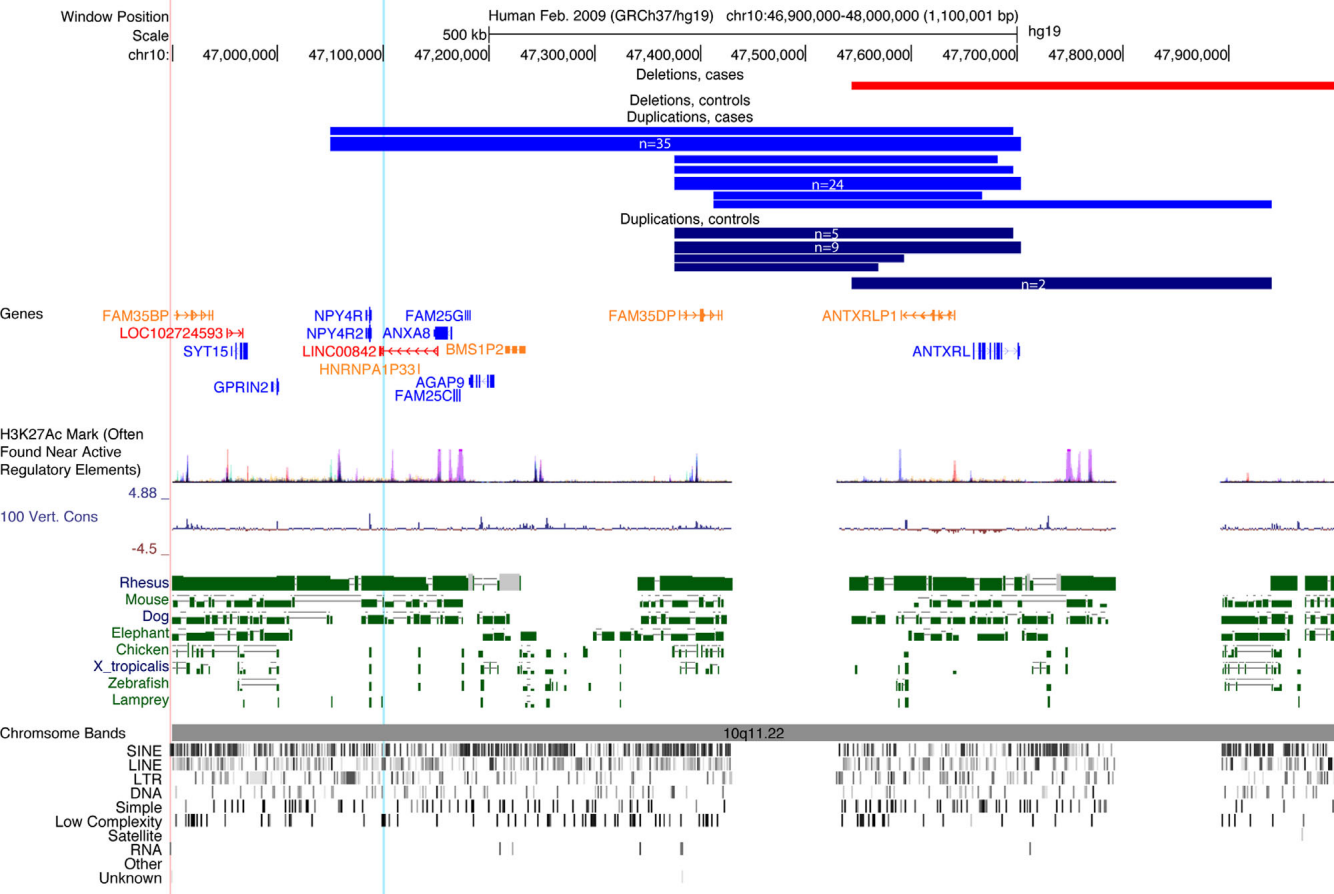
Chromosome 10 duplication significantly enriched in esotropia cases. UCSC genome browser plot showing the region of duplication on 10q11.22 (10:47049547-47703870). Duplications across the full 700kb were present in 36 cases (*light blue*, n indicates number with each set of breakpoints). A smaller, ∼300-kb duplication was present in 28 additional cases and 18 controls (*dark blue*). A nearby deletion was present in one case (*red*). RefSeq genes are listed underneath. Protein coding genes are in *blue*, long noncoding RNA genes are in *red*, and noncoding RNAs are in *orange*. H3K27Ac mark indicates several putative regulatory regions fall within the duplication. This area is not well conserved over 100 vertebrates. There is some conservation with monkey, but very little with other animals. The *blue vertical line* indicates the position of the ddPCR probe used to confirm the duplication. The gap in the annotations indicates an unmappable area of the reference genome, usually because it is highly repetitive or of low complexity. At bottom are indicated repeats in the region identified by RepeatMasker: SINE, short interspersed nuclear elements; LINE, long interspersed nuclear elements; LTR, long terminal repeat elements; DNA, DNA repeat elements; SIMPLE, microsatellites, low complexity repeats, satellite repeats, RNA repeats, and other repeats.

A significant 654kb locus was identified on chromosome 10q11.22 (hg19, chr10:47049547-47703870, *P*_corr_ = 1 × 10^−^^6^), spanning the protein-coding genes *NPY4R*, *NPY4R2*, *ANXA8*, *FAM25C*, *FAM25G*, *AGAP9*, and *ANTXRL*, the lncRNA *LINC00842*, and pseudogenes *HNRNPA1P33*, *BMS1P2*, *FAM35DP*, and *ANTXRLP1*, which were all also identified with the gene based test (*P*_corr_ = 1 × 10^−^^6^, for each). A CNV at this locus was present in 64 cases (4.0%) and 18 controls (0.4%), corresponding to a substantially increased esotropia risk (OR 8.96; 95% CI 5.4–14.9). Notably, the duplication in 36 esotropia cases and 0 controls spanned the full 654 kb, whereas 28 cases and 18 controls had a smaller ∼300 kb duplication. *NPY4R*, *NPY4R2*, *LINC00842*, *HNRNPA1P33*, *ANXA8*, *FAM25C*, *FAM25G*, *AGAP9*, and *BMS1P2* are within the portion of the duplication seen only in the esotropia cases. The presence of the smaller duplication was associated with an increased esotropia risk (OR 3.918; 95%CI 2.2-7.2), indicating that the association is not a result of only the larger, extended duplication. The region has several putative regulatory elements but is poorly conserved and has multiple repetitive elements (Fig. 5).

Each of the significant CNVs was validated in affected cases by ddPCR. All patients with chromosome 2 and 4 duplications were validated, and all patients with the larger chromosome 10 duplication were validated, because the probe location is within the region unique to esotropia patients. Overall, 114 cases (7%) and 28 controls (0.7%) had one of the three duplications. No case nor control had more than one of the duplications.

We repeated association testing after removing related individuals within the esotropia cohort (see methods), leaving 1379 cases. The same three duplications were again significant to *P* = 1 × 10^−^^6^, by both the breakpoint test and gene test, indicating that our results were not driven by relatedness of our cases.

### Insertion and Breakpoint Analysis

To determine whether the duplications were tandem or interspersed and to identify the breakpoints, three cases with each duplication were chosen for WGS. Sequencing all individuals was not feasible, so we chose several unrelated individuals with each duplication who harbored different predicted breakpoints based on SNP calling. For the chromosome 2 duplication we sequenced one infantile and two nonaccommodative esotropia participants. For the chromosome 4 duplication we sequenced one accommodative, one partially accommodative, and one nonaccommodative esotropia participant. For the chromosome 10 duplication we sequenced three nonaccommodative esotropia participants.

**Figure 6.**
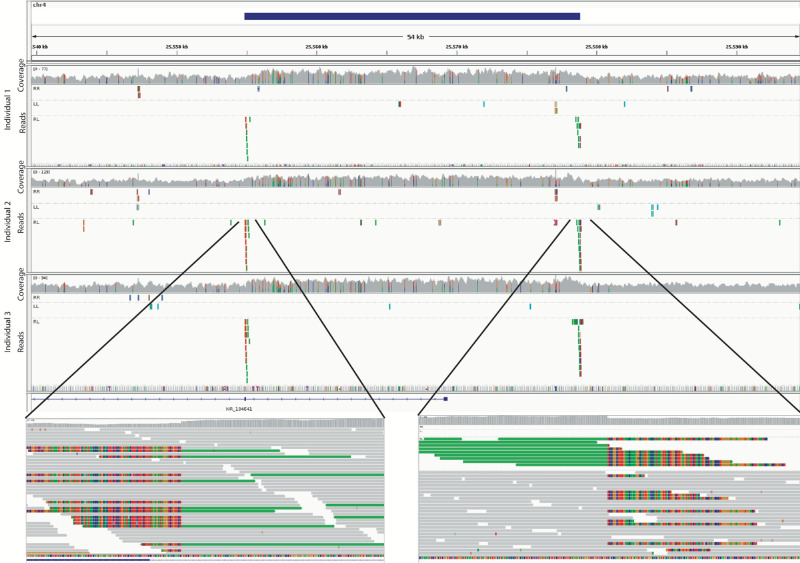
Breakpoints of chromosome 4 duplication. Whole genome sequences from three unrelated individuals with esotropia who harbor the chromosome 4 duplication show increased sequence coverage across the duplication and split reads at the breakpoints. *Top*: Schematic of chromosome 4 region of duplication. *Middle*: Images from integrated genome viewer for each of three individuals. Coverage is indicated for each base pair by the height of the gray bar, split reads are shown in *red* and *green* below. Green reads indicate that the paired read maps further away than expected. *Bottom*: individual reads are shown across the breakpoints. The split reads (colored by base-pair that does not map to the reference sequence) were mapped back to indicate this is a tandem duplication. The left breakpoint (hg38: 4:25,554,985) is just upstream of exon 2 of LOC101929. The right breakpoint (hg38: 4:25,578,843) is in an intergenic region.

Despite the SNP prediction of different breakpoints, the three individuals with the chromosome 4 duplication all harbored a tandem duplication with breakpoints at chr4:25,554,985 and chr4:25,578,843 (hg38, which correspond to hg19:chr4:25,556,607 and chr4:25,580,465). Split-reads were readily identified that span the breakpoints, and sequencing coverage was higher across the area of duplication. Exon 1 of *LOC101929161* is included in the duplication and the breakpoint is just upstream of the exon 2 junction (Fig. 6). The chromosome 2 and chromosome 10 duplications were in areas of the genome with multiple repetitive elements and poor mapping of short sequencing reads. We therefore could not identify definitive breakpoints for these two duplications in these individuals, nor determine whether the duplications were tandem or interspersed.

### Esotropia Subtypes

**Figure 7.**
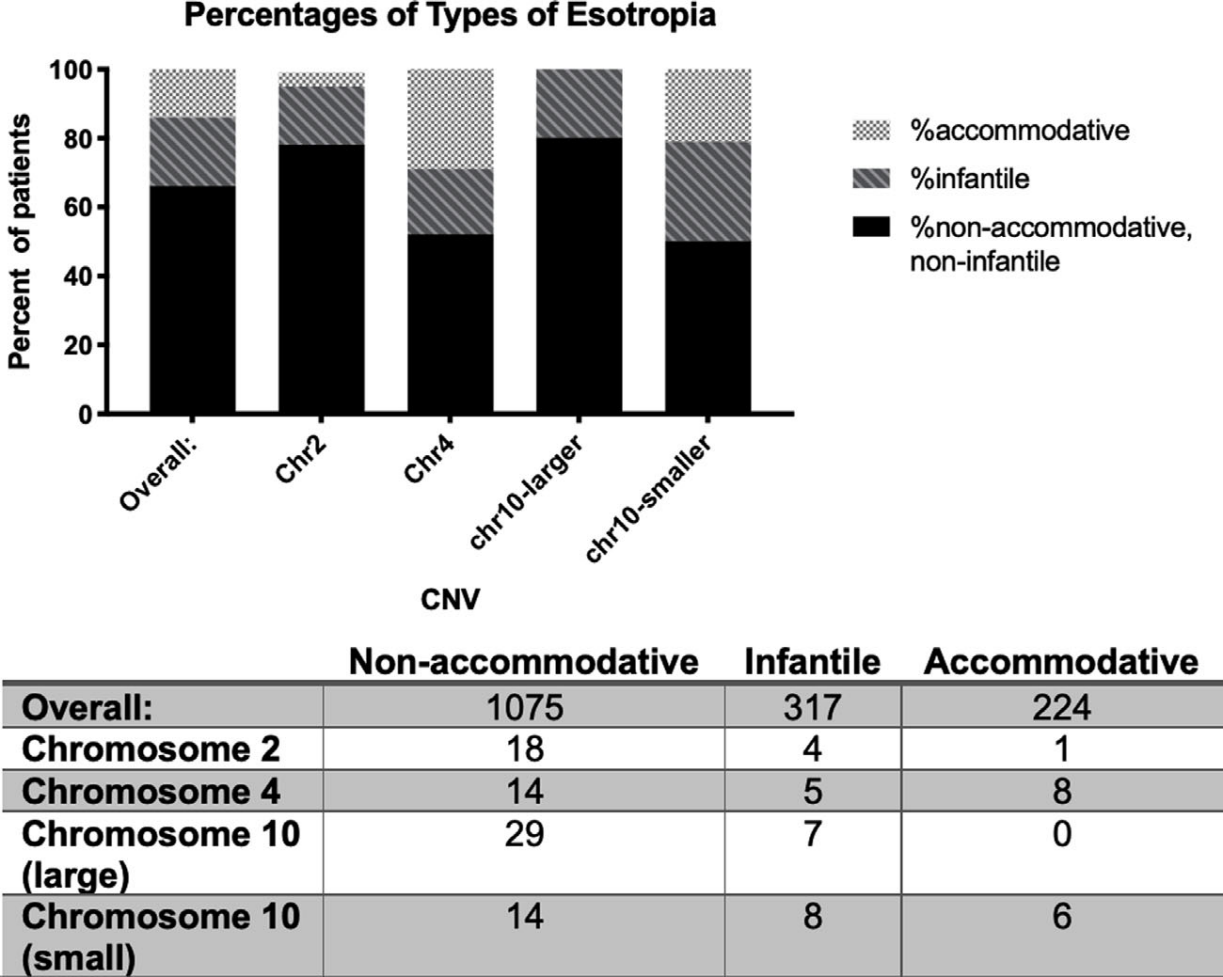
Distribution of subtypes of esotropia. The overall esotropia cohort consists of 14% accommodative (*light grey checkerboard*), 20% infantile (*dark gray stripes*), and 66% nonaccommodative, noninfantile (*black*). Accommodative esotropia is underrepresented in the chromosome 2 and larger chromosome 10 duplications, and overrepresented in the chromosome 4 duplication. *P* = 0.069, chi square. Numbers of participants in each group are provided in the table.

To determine whether these duplications were associated with subtypes of esotropia, we compared the proportion of participants in the cohort with accommodative, infantile, or nonaccommodative esotropia (as defined above) to the proportion with each duplication. In the full cohort, 1075 (66.5%) participants were classified as nonaccommodative, 317 (19.6%) as infantile, and 224 (13.9%) as accommodative. Although the numbers are small, the distribution of subtypes differs significantly between the duplications (chi square 17.74, degrees of freedom 6, *P* = 0.0069). Accommodative esotropia is underrepresented in patients with the chromosome 2 duplication (only 1 [4%] of individuals with the chromosome 2 duplication had accommodative esotropia), and absent from patients with the larger chromosome 10 duplication. By contrast, accommodative esotropia is overrepresented among patients with the chromosome 4 duplication (eight cases [29.6%] and the smaller chromosome 10 duplication (six cases (21.4%) (Fig. 7).

## Discussion

We demonstrate a role for CNVs in the risk for esotropia, a disorder with poorly understood pathophysiology. We observe a greater global burden of total rare CNV length, and report three recurrent rare duplications that significantly increase risk.

The chromosome 4 duplication includes exon 1 of *LOC101929161*, a lncRNA of unknown function encompassing 4 exons. This RNA is exclusive to primates, with no homology in mice. In published RNASeq data, expression is primarily in lung and digestive system.^50^^,^^51^ Duplicating one exon could alter the conformation of the RNA molecule, affecting its affinity for its binding partners. Alternately, the duplication could change the 3D chromatin structure, affecting the topographically associated domains and thus regulation of nearby genes.

The chromosome 2 duplication encompasses one lncRNA (*CYTOR*) and two overlapping microRNAs (miR4435-1 and miR4435-2). CYTOR is broadly expressed in fetal and adult tissues, with low levels in adult and fetal brain,^50^^,^^52^ and is overexpressed in cancer cells.^53^ The two microRNAs are single exons and are presumed to regulate translation of other genes. There are no homologous genes or microRNAs in mouse. The duplicated region contains multiple putative regulatory regions, and duplication of these could alter expression of their target genes or genes near the insertion site.

The chromosome 10 duplication includes 12 genes, of which only a few have known functions. *NPY4R* and *NPY4R2* encode neuropeptide Y receptors; neuropeptide Y is a gut-brain peptide which modulates multiple physiologic processes, including feeding behavior and anxiety.^54^ *ANXA8* encodes annexin 8, one of a family of Ca^++^ effector molecules that regulate EGF receptor localization and activity.^55^ *ANTXRL*, *FAM25C*, *FAM25G*, and *AGAP9* are protein-coding genes of unknown function. *LINC00842* is a lncRNA of unknown function. *HNRNPA1P33*, *FAM35DP*, *ANTXRLP1*, and *BMS1P2* are pseudogenes.

None of the genes involved in the duplications suggest an obvious pathologic mechanism for strabismus, but study of their developmental expression patterns and functions may lead to further insights into strabismus. The genetic loci identified as strabismus risk factors through GWAS, WRB^21^ and NPLOC4-TSPAN10-PDE6G,^22^ similarly do not have obvious roles in strabismus pathology.

WGS in three individuals with the chromosome 4 duplication showed the duplication is tandem and defined the breakpoints. Although these individuals are unrelated, the breakpoints are identical. These particular breakpoints may be a “hotspot” for new duplications, or these individuals may share an ancestral haplotype that includes this duplication and confers risk for esotropia.

The breakpoints could not be definitively identified in the individuals with chromosome 2 and 10 duplications, because the breakpoint regions are in areas of the genome with highly repetitive sequence. Mapping reads and identifying split reads in these areas is difficult using short-read next generation sequencing, because 100 to 150 base pair reads of repetitive sequence map to multiple locations in the genome. This hinders CNV calling by WGS. By contrast, SNP calling uses SNPs present across the region, and these CNVs were validated with ddPCR. Unfortunately, repetitive genomic areas are those most likely for insertion and deletion events to occur. This is a problem throughout the field of genetics, which may be solved in the future by long read sequencing.

A limitation of using publicly available control datasets is that individuals with strabismus, especially a history of treated childhood strabismus, may be included in our control set. This, however, strengthens our findings, because some of the control individuals with these duplications may have strabismus. Similarly, strabismus patients may be included in public databases of “healthy” individuals, making comparisons to public databases of CNVs difficult to interpret. DGV reports structural variation present in healthy individuals, from studies that called CNVs using differing algorithms and genotyping platforms. A similar duplication on chromosome 4 has a frequency of 0.34% in DGV,^56^ similar to the 0.2% rate in our controls. On chromosomes 2 and 10, somewhat larger duplications have frequencies of 1.58% and 1.74%, respectively, much higher than in our control population (0.2% and 0.4%). This may reflect differences in CNV calling algorithms and genotyping platforms, quality control measures, or populations included. Using these control frequencies, the chromosome 10 duplication remains significant whereas the chromosome 2 duplication does not. Because our cases and controls were called using identical parameters, they provide a more valid comparison.

Although we have one of the largest and most accurately phenotyped esotropia cohorts, it remains underpowered to detect extremely rare CNVs or those of moderate effect. Although we show an association between these rare duplications and esotropia subtypes, our numbers are too small to draw definitive conclusions. Whether these duplications are more prevalent in exotropia, indicating a genetic predisposition to strabismus generally, rather than esotropia specifically, remains to be explored.

Large CNVs cause many genetic syndromes that include strabismus, including Down syndrome (duplication chromosome 21),^57^^–^^59^ Williams-Beuren syndrome (deletion 7q11.23),^60^ and deletion of 10q26.^61^ CNVs have also been reported in patients with Duane syndrome, a form of paralytic strabismus.^62^ Interestingly, large (>5Mb) duplications of 10q11, which encompass the 654kb region we report, cause 10q duplication syndrome which includes developmental delay, dysmorphic features, and, in 5/8 cases, strabismus.^63^ A male with a 4.5Mb duplication of 4p15.2, which encompasses the 23kb duplication we report here, was reported to have developmental delay, congenital heart disease and strabismus.^64^ The participants in this study all had nonsyndromic strabismus, and there are no specific syndromes associated with the precise duplications we identified.

We provide here the first evidence that CNVs contribute to genetic risk in nonsyndromic esotropia. Further research into the functional consequences of these duplications will hopefully increase our understanding of the pathophysiology of strabismus.

## Appendix

Members of the Strabismus Genetics Research Consortium:

USA: Anna Baglieri, Brenda Barry, Sarah Bekele, Sarah E. Breau, Kimberley Chan, Frances Corkin, Linda R. Dagi, Alexandra Elliott, Janet Esligar, Caroline Fang, Anne B. Fulton, Gena Heidary, Suzanne Johnston, Melanie Kazlas, Danielle M. Ledoux, Richard L. Levy, Iason S. Mantagos, Kathryn B. Miller, Monte Mills, Darren Oystreck, Christina S. Petersen, Robert A. Petersen, Carrie E. Pierce, Aparna Raghuram, Richard Robb, Josephine C. Sandoval, Sonia Sethee, Ankoor S. Shah, Lois E.H. Smith, Melissa Toffoloni, Deborak K. Vanderveen, Sarah Whitecross, Rupa K. Wong, Carolyn Wu.

Australia: Julie Barbour, Linda Clarke, Joanne C. Dondey, Maree Flaherty, John Grigg, Kate Hanman, Michael Haybittel, Robyn V. Jamieson, Lisa S. Kearns, Lionel Kowal, Geoffrey C. Lam, Troy Lim Joon, John McKensie, Loren Rose, Jonathan B. Ruddle, Lindsey Scotter, Neil E. Sinclair, Colleen Wilkinson, Robin Wilkinson.

United Kingdom: Viral Sheth, Mervyn G. Thomas.
